# Unusual Cause of a Cardiac Arrest in a Former African American Collegiate Athlete

**DOI:** 10.7759/cureus.31645

**Published:** 2022-11-18

**Authors:** James P Gillen, Diego Riveros, Leila Azari

**Affiliations:** 1 Internal Medicine, Morsani College of Medicine, Tampa, USA

**Keywords:** apical cardiomyopathy, athlete, yamaguchi, hypertrophic cardiomyopathy, cardiac arrest

## Abstract

Yamaguchi-variant cardiomyopathy is an underreported but significant cause of cardiac arrest among athletes. We studied the hospital course of one patient who arrived at the emergency department after a sudden cardiac arrest while playing a recreational basketball game. We used the electronic medical record (Epic) to follow the notes, labs, imaging, and procedures that were performed. Although a rare disease, Yamaguchi syndrome should not be overlooked when working up a patient who has suffered a sudden cardiac arrest. Proper knowledge of automatic external defibrillators and basic cardiopulmonary resuscitation principles can have a significant positive impact, and the importance of these interventions should not be overlooked in patients with a sudden cardiac arrest.

## Introduction

Sudden cardiac death (SCD) during exercise is a significant cause of mortality worldwide among athletes. A recent study estimated rates of SCD fall between one in 40,000 and one in 80,000 [1]. Cardiac arrest in athletes has a broad differential which can be grouped as inherited and acquired, with both groups further divided into structurally normal and abnormal hearts [2]. Structurally normal inherited conditions include channelopathies such as Brugada syndrome, accessory pathways such as Wolf-Parkinson-White, and long QT syndrome [3]. Structurally abnormal conditions include hypertrophic cardiomyopathy (HCMO) and arrhythmogenic right ventricular dysplasia. Inherited conditions tend to affect younger athletes with acquired conditions such as atherosclerotic coronary artery disease affecting older athletes [4-6]. As the most common inherited cardiac abnormality, HCMO is present in one in 200 in the general population [7]. As these patients are generally assumed to be young and healthy, the societal and emotional impact of HCMO cannot be overstated [7]. Discussing a rare variation of this disease and its presentation is, therefore, an important exercise for medical providers caring for these athletes.

## Case presentation

**Figure 1:**
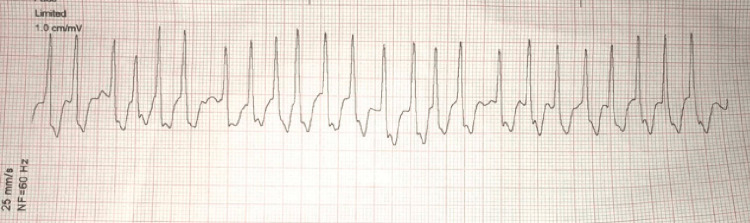
Rhythm strip obtained by emergency medical service after two automatic external defibrillator shocks prior to arrival at the emergency department.

A 45-year-old African American male with a medical history significant for hypertension on lisinopril per family presented to Tampa General Hospital emergency department (ED) status after cardiac arrest. The patient reportedly was playing basketball at 0630 when he suddenly collapsed and was found to be pulseless. Cardiopulmonary resuscitation (CPR) was initiated immediately by his fellow basketball players including a physician on the scene. The patient received two automatic external defibrillator (AED) shocks with a return of spontaneous circulation. Ventricular tachycardia was the first rhythm described by emergency medical service followed by atrial fibrillation with rapid ventricular response seen on the rhythm strip en route to the hospital (Figure 1).

**Figure 2:**
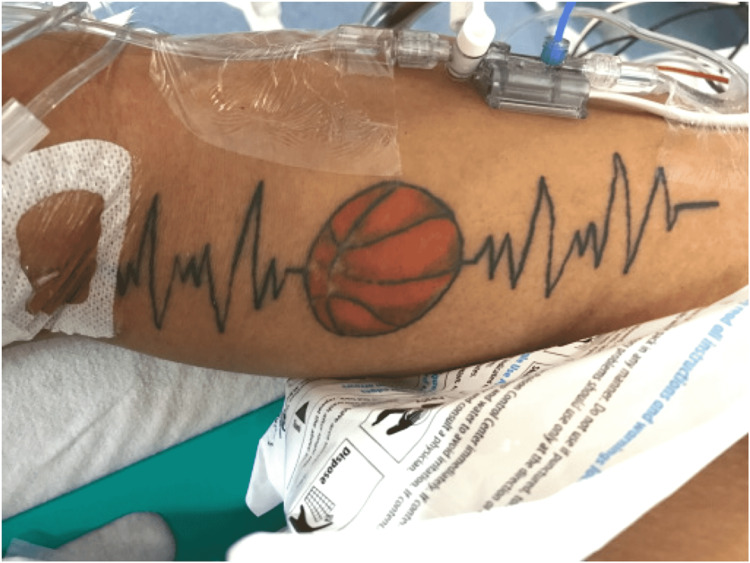
Tattoo found on patient’s arm upon presentation to the emergency department (with permission from the patient).

The patient spontaneously converted to sinus tachycardia prior to a 150 mg bolus of amiodarone. The patient was unconscious on arrival to the ED where rapid sequence endotracheal intubation was performed using succinylcholine and etomidate without incident. A tattoo was seen on the patient’s upper extremity of a basketball with an abnormal rhythm strip on either side of unknown significance (Figure 2).

**Figure 3:**
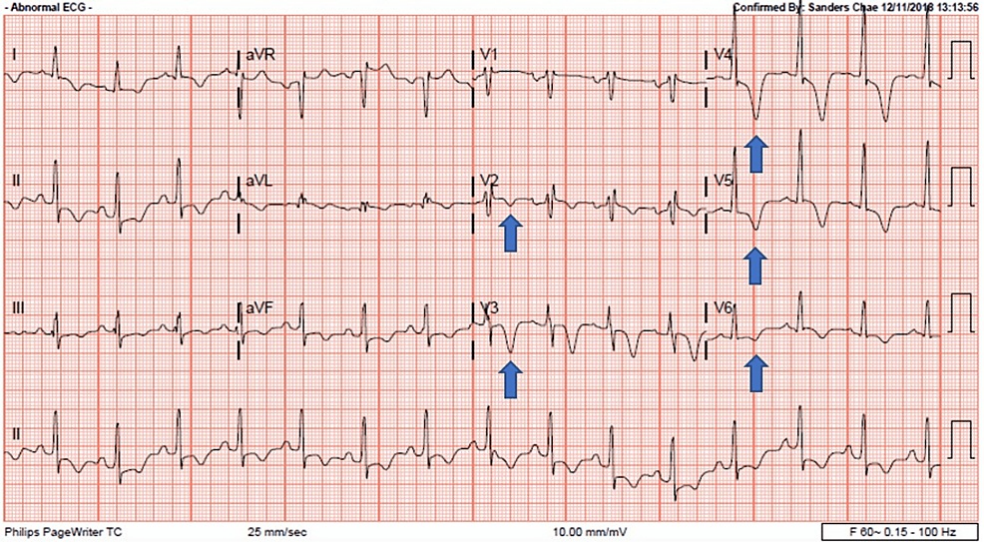
Initial electrocardiogram obtained upon arrival to the emergency department after spontaneous conversion to sinus rhythm. Inverted T waves seen in the limb leads along with deep inverted T waves in V2-V6.

An electrocardiogram (ECG) showed left atrial and possible bi-atrial enlargement demonstrated by sharp P wave in lead II and semi-bifid P wave in V1 with inverted T waves in the limb leads along with deep inverted T waves in V2-V6, non-specific intraventricular conduction delay, and voltage criteria for left ventricular hypertrophy (LVH) without ST-elevation myocardial infarction (Figure 3). A bedside echocardiogram in the ED was performed and confirmed the suspicion of HCMO, but the atypical apical variant, aka Yamaguchi syndrome.

**Figure 4:**
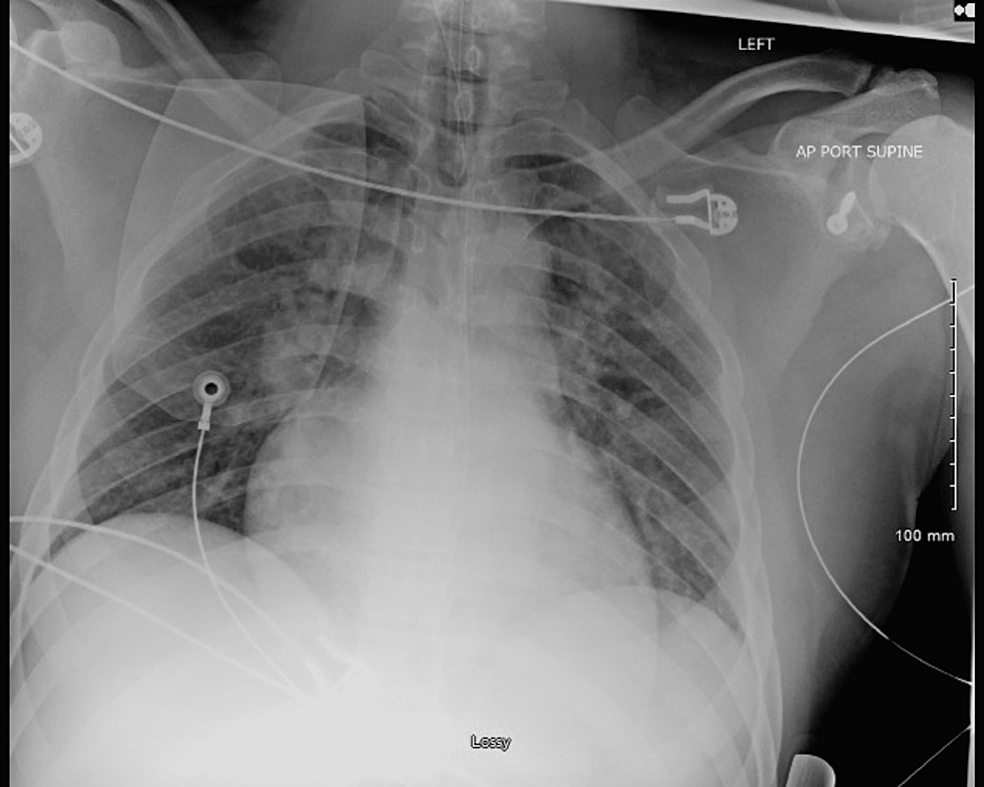
Chest X-ray post-intubation in the emergency department, notable for mild central pulmonary vascular congestion.

In the ED, the patient was hypotensive requiring aggressive fluid resuscitation with lactated ringers along with phenylephrine bolus and drip. The family arrived and confirmed his medical history of hypertension and denied a family history of SCD. Labs were notable for arterial blood gas pH - 7.36/pCO_2_ - 30/PO_2_ - 400/HCO_3_ - 17 while on a 100% non-breather mask with K 3.6 and a lactate of 7.7 drawn prior to intubation. The comprehensive metabolic panel was notable for albumin/globulin 18 and serum creatinine of 1.6. Brain natriuretic peptide was mildly elevated at 103, and troponin was negative. Chest radiography (CXR) was remarkable for mild central pulmonary vascular congestion (Figure 4).

**Figure 5:**
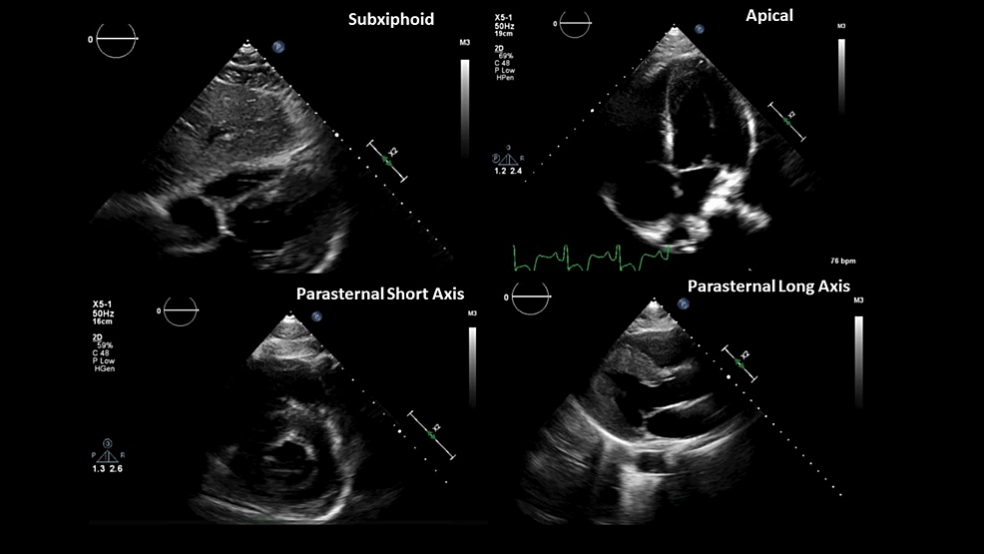
Two-dimensional echocardiogram showing severe concentric left ventricular hypertrophy.

A cooling protocol was initiated with a goal temperature of 32°C using propofol and fentanyl for sedation with cisatracurium as a paralytic. Sinus bradycardia down to the high 30s was noted during the cooling period that resolved after re-warming on day 3. The patient did have non-sustained runs of atrial fibrillation with a rapid ventricular response (AF/RVR) upon rewarming. Transesophageal echocardiogram found severe concentric LVH, with relatively severe hypertrophy of mid to apical myocardium and no evidence of intraventricular or left ventricular outflow tract (LVOT) obstruction (Figure 5, Video 1). The estimated ejection fraction (EF) was in the range of 60% to 65%. There were no regional wall motion abnormalities. Features were consistent with a pseudonormal left ventricular filling pattern, with concomitant abnormal relaxation and increased filling pressure and grade 2 diastolic dysfunction.

**Video 1:** Two-dimensional echocardiogram showing severe concentric left ventricular hypertrophy.

**Figure 6:**
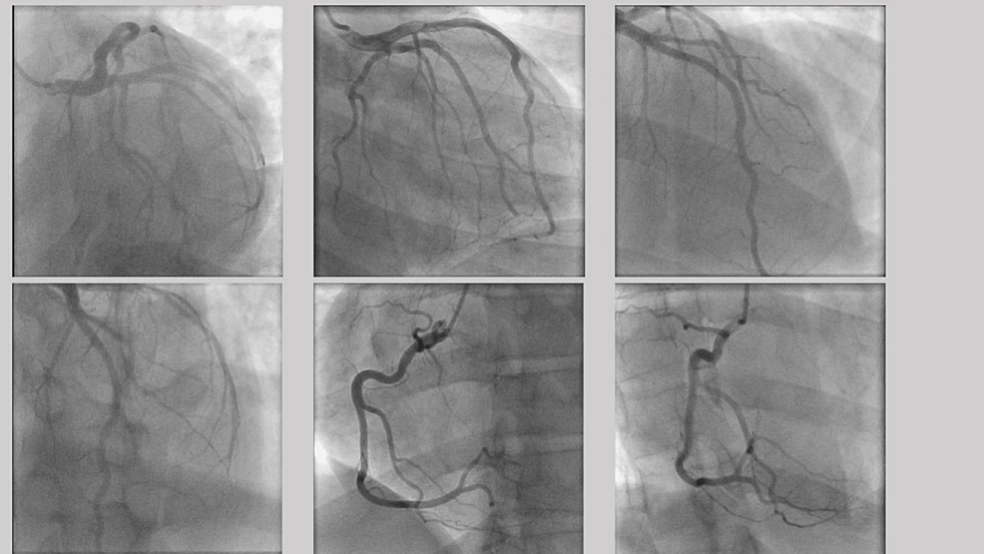
Left heart catheterization with left ventricular angiography.

The mitral valve had evidence of systolic anterior motion, with mild mitral regurgitation noted. The main pulmonary artery was normal-sized with systolic pressure within the normal range, estimated to be 26 mmHg. The inferior vena cava was dilated with blunted respire-phasic diameter changes (<50%), consistent with elevated central venous pressure (Figure 5, Video 1). Left heart cardiac catheterization found no obstructive coronary artery disease without significant LVOT gradient noted and mildly increased left ventricular end-diastolic pressure (Figure 6, Video 2). Severe apical LVH with hypercontractility was noted.

**Video 2:** Left heart catheterization with left ventricular angiography.

**Figure 7:**
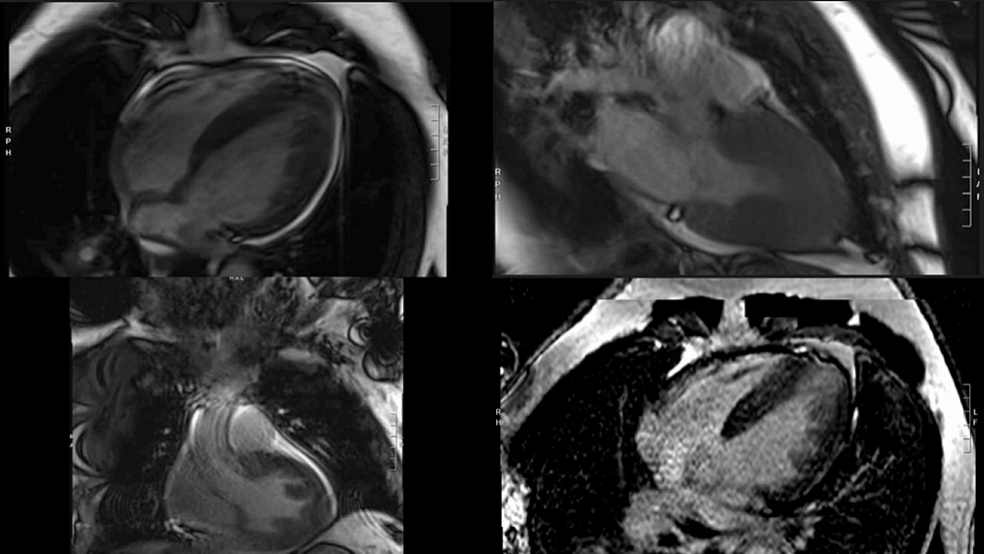
Cardiac magnetic resonance imaging with and without contrast.

Cardiac magnetic resonance imaging (MRI) with and without contrast found a normal overall left ventricular size with an EF of 80% by Simpson’s method (Figure 7, Video 3). Global left ventricular function was hyperdynamic without regional wall motion abnormality. Asymmetric eccentric LVH was noted, predominantly involving the apex and mid ventricle with maximal thickness measuring up to 16.0 mm. There was no systolic anterior motion of the mitral valve or obstruction (Video 3). There was no perfusion deficit or delayed gadolinium enhancement. The right ventricle was normal without enhancement, with normal bi-atrial size along with a small pericardial effusion.

**Video 3:** Cardiac magnetic resonance imaging with and without contrast.

The patient was warmed on day 3 of his hospitalization. Neurologically, he was intact except for residual memory loss of the morning of his cardiac arrest. A computed tomography scan of the brain obtained in the ED was normal. The patient was initially hypotensive during his critical care unit stay requiring fluid resuscitation and phenylephrine drip. After re-warming and extubation, hypertension developed and was treated with increasing doses of metoprolol succinate. An implantable cardioverter-defibrillator (ICD), St. Jude single-chamber (RV) generator (Frotify Assura, using a Medtronic 62 cm RV lead), was placed prior to discharge.

The patient had a cardiogenetics consult as an outpatient without abnormality found on his personal genetic analysis. His two daughters had negative echocardiograms and were genetically tested as well. Genetic testing was negative for HCMO for both, typical for families of Yamaguchi syndrome patients.

The patient was switched from metoprolol to diltiazem in the clinic secondary to the usual beta-blocker adverse effects. ICD was episodically interrogated in the clinic without arrhythmia seen. The patient was advised of the importance of maintaining hydration, heart rate control, and balancing lifestyle features to a steady state. Exercising was not completely prohibited, but he was told to avoid strenuous activity and dehydration at all costs. The patient started wearing a bio-feedback device as advised to ensure the heart rate does not exceed 140-150 beats per minute while playing non-strenuous basketball. Six months after his cardiac arrest, the patient is playing basketball four times a week without any symptoms or limitations. When asked what he was trying to say with his tattoo of a basketball surrounded by a rhythm strip, he said “basketball is my life.”

## Discussion

We presented a case of a rare apical variant of HCMO (avHCM), namely, Yamaguchi syndrome. Named after Yamaguchi in 1979, this apical variant has an autosomal dominance familial inheritance similar to the typical variant HCMO [8,9]. Yamaguchi syndrome has been shown to be rarely found in those of African descent [10]. For all cases of HCMO in Japan, however, Yamaguchi syndrome is estimated to be present in 15% of cases, whereas in the US, the apical variant only accounts for 3% [10]. As such, Yamaguchi syndrome presents a diagnostic challenge for healthcare practitioners caring for these patients in the US.

The pattern of hypertrophy in avHCM affects the pathophysiology of this disease. Due to the hypertrophy present mostly in the apex, there is generally no LVOT obstruction. Typical features of this disease include fourth heart sound, left ventricle spade-like formation found on imaging, large precordial negative T waves, and apical wall motion abnormalities [11]. Different imaging modalities exist to aid in the diagnosis with the most accurate being cardiac MRI [12].

Patients with Yamaguchi syndrome were typically thought to have a benign course with mild to no symptoms. One study found that the average age of presentation was 41 years, with a 95% survival rate at 15 years and cardiac mortality of 1.9% [11]. However, as was present in this case, patients may develop severe complications such as arrhythmias and myocardial infarctions. One-third of patients in the beforementioned study were found to develop one or more major morbid events including atrial fibrillation (12%) and myocardial infarction (10%). While it has been thought to have a benign course, the rarity of Yamaguchi syndrome lends to poor data collection, and further insight into the prevalence and disease course of this entity is paramount.

## Conclusions

We present an unusual cause of cardiac arrest in a 45-year-old African American former Division 1 basketball player who had never previously had an ECG or echocardiogram during previous medical screening examinations. He recovered completely with only minimal memory deficit of the event. We propose very strongly that all athletes, whether at junior high school, high school, or collegiate levels, receive an ECG and screening echocardiogram prior to initiation of competition. We also agree that AEDs should be placed generously throughout the general and athletic community. Personnel working near AEDs must receive periodic and scheduled teaching with hands-on training and simulation.
